# Effects of Bromocriptine on Glucose and Insulin Dynamics in Normal and Insulin Dysregulated Horses

**DOI:** 10.3389/fvets.2022.889888

**Published:** 2022-05-31

**Authors:** Caroline M. M. Loos, Kristine L. Urschel, Eric S. Vanzant, Erin L. Oberhaus, Adam D. Bohannan, James L. Klotz, Kyle R. McLeod

**Affiliations:** ^1^Department of Animal and Food Sciences, University of Kentucky, Lexington, KY, United States; ^2^School of Animal Sciences, Louisiana State University, Baton Rouge, LA, United States; ^3^Forage-Animal Production Research Unit, Agricultural Research Service, United States Department of Agriculture, Lexington, KY, United States

**Keywords:** bromocriptine, horse, glucose, insulin, lipids

## Abstract

The objectives of the study were to study the effects of the synthetic ergot alkaloid (EA), bromocriptine, on glucose and lipid metabolism in insulin dysregulated (ID, *n* = 7) and non-ID (*n* = 8) mares. Horses were individually housed and fed timothy grass hay and two daily concentrate meals so that the total diet provided 120% of daily DE requirements for maintenance. All horses were given intramuscular bromocriptine injections (0.1 mg/kg BW) every 3 days for 14 days. Before and after 14 days of treatment horses underwent a combined glucose-insulin tolerance test (CGIT) to assess insulin sensitivity and a feed challenge (1 g starch/kg BW from whole oats) to evaluate postprandial glycemic and insulinemic responses. ID horses had higher basal plasma concentrations of insulin (*P* = 0.01) and triglycerides (*P* = 0.02), and lower concentrations of adiponectin (*P* = 0.05) compared with non-ID horses. The CGIT response curve showed that ID horses had slower glucose clearance rates (*P* = 0.02) resulting in a longer time in positive phase (*P* = 0.03) and had higher insulin concentrations at 75 min (*P* = 0.0002) compared with non-ID horses. Glucose (*P* = 0.02) and insulin (*P* = 0.04) responses to the feeding challenge were lower in non-ID compared to ID horses. Regardless of insulin status, bromocriptine administration increased hay intake (*P* = 0.03) and decreased grain (*P* < 0.0001) and total DE (*P* = 0.0002) intake. Bromocriptine treatment decreased plasma prolactin (*P* = 0.0002) and cholesterol (*P* = 0.10) and increased (*P* = 0.02) adiponectin concentrations in all horses. Moreover, in both groups of horses, bromocriptine decreased glucose clearance rates (*P* = 0.02), increased time in positive phase (*P* = 0.04) of the CGIT and increased insulin concentrations at 75 min (*P* = 0.001). The postprandial glycemic (*P* = 0.01) and insulinemic (*P* = 0.001) response following the oats meal was lower after bromocriptine treatment in all horses. In conclusion, in contrast to data in humans and rodents, bromocriptine treatment reduced insulin sensitivity in all horses, regardless of their insulin status. These results indicate that the physiological effects of EA might be different in horses compared to other species. Moreover, because bromocriptine shares a high degree of homology with natural EA, further investigation is warranted in horses grazing endophyte-infected grasses.

## Introduction

Insulin dysregulation (ID), characterized by basal hyperinsulinemia and an exacerbated insulin response to an oral or intravenous glycemic challenge (1), remains a prevalent clinical problem in the equine industry. In particular, its association with laminitis (2), one of the most detrimental clinical conditions seen in veterinary practice, continues to push scientists toward the development of treatment strategies for equine metabolic diseases. Despite significant progress in our understanding of the pathophysiological mechanisms underlying the onset of laminitis, prognosis of affected animals remains poor. Therefore, preventative measures, including efforts to mitigate ID, should be the primary focus in the management of predisposed horses. While a variety of commercially available supplements and drugs claim to improve insulin sensitivity anecdotally, few science-based pharmaceutical treatments currently exist in horses.

Bromocriptine, a synthetic ergot alkaloid and dopamine receptor agonist has been shown to improve insulin sensitivity in both healthy and ID humans and rodents and is used as a drug to lower blood glucose levels in diabetic individuals (3, 4). Although the exact mechanism by which bromocriptine improves insulin sensitivity is not fully elucidated, its beneficial effects have been attributed to restoration of insulin resistance-associated changes in dopaminergic activity and lowering prolactin levels (5).

In horses, synthetic ergot alkaloids, such as those found in the drug pergolide, are already used to treat symptoms associated with Equine Cushing's disease. The anecdotal use of this drug to treat ID in horses has spurred research interest to look at the effects of alkaloid compounds on insulin metabolism. Oral administration of pergolide mesylate for >3 months in horses with pituitary pars intermedia dysfunction (PPID) did not alter glucose and insulin metabolism (6). It is important to note this was an uncontrolled study with use of privately owned horses and the dosage of pergolide administration and management varied between horses, making conclusive inferences difficult. Repeated intramuscular injections with another dopamine receptor agonist, cabergoline, for 45 days did not alter the insulin response to an intravenous glucose infusion (7). However, that study did not assess glucose clearance rate after exogenous glucose and insulin infusion or the insulin response to an oral glycemic challenge. It is known that hyperinsulinemia can be the end-stage manifestation of a disruption at different stages of insulin metabolism (1). Therefore, dynamic testing through both oral and intravenous glycemic challenges is recommended for a more complete evaluation of any potential changes in insulin metabolism. Moreover, while cabergoline and bromocriptine are both dopamine agonists, their metabolic effects may differ due to pharmacokinetic and physiochemical differences between the two compounds (8). In fact, studies in humans suggest that bromocriptine treatment specifically promotes glucose disposal after an oral glucose load, potentially acting as a unique postprandial “weighted” insulin sensitizer (9). No data currently exists regarding the effects of bromocriptine treatment on glucose homeostasis and insulin sensitivity in horses. Bromocriptine also shares a high degree of homology with ergovaline, the predominant ergot alkaloid found in endophyte-infected tall fescue, a common grass species in equine pasture and hay. Therefore, evaluation of the effect of bromocriptine on insulin sensitivity does not just have implications for pharmaceutical treatment but is also highly relevant for grazing horses.

The objectives of the current study were to evaluate the effects of bromocriptine on glucose and insulin dynamics following a combined glucose insulin tolerance test and a high starch feeding challenge in ID and non-ID horses.

## Materials and Methods

### Animals and Housing

Sixteen mature mares of mixed breed were selected from the herd located at the University of Kentucky Veterinary Science Department research farm. As several research horses were donated to the University, the exact breed for several horses was unknown but included Thoroughbreds, Standardbreds, Arabians, Tennessee Walking horses and crosses thereof. Candidate horses were identified based on their insulin status which was determined by an oral sugar test (OST) as previously described (10). Briefly, two blood samples were collected *via* jugular venipuncture before and 60 min after administration of 0.15 mL/kg body weight (BW) of light corn syrup (Karo, ACH Food Companies, Chicago, IL). Horses had access to pasture prior to the OST procedures but did not receive their morning concentrate meal. Based on the recommended criteria published by the Equine Endocrinology Group (EEG)^1^ horses were defined as being insulin dysregulated (ID) when plasma insulin concentrations were >20 and >45 μIU/mL at baseline and 60 min post OST, respectively. Based on these criteria, eight ID and eight non-ID horses were selected for the study (Table 1). Due to medical reasons, unrelated to the experiment, one horse in the ID group had to be removed from the study, resulting in seven ID horses completing the experiment. To exclude horses with pituitary pars intermedia dysfunction (PPID), basal plasma adrenocorticotropic hormone (ACTH) levels were determined from a morning blood sample collected during the OST screening procedures. All horses had ACTH concentrations <50 pg/mL with exception of one horse in the ID group, who had an ACTH value of 82 pg/mL. Based on the 2019 recommendations by the EEG for fall months,^2^ basal ACTH values <50 or 50–100 pg/mL are considered negative and equivocal for PPID, respectively. As this horse showed no other clinical signs of PPID, the decision was made to include her in the study. There was no indication that this horse was an outlier for any of the variables measured and her data were included in the final analysis.

**Table 1:** Insulin concentrations before and 60 min post oral sugar test, initial body weight and body condition score, and age for the non-ID and ID horses.

**Phenotypic measure**	**Non-ID**	**ID**	***P*-values**
Insulin OST 0 min (μIU/mL)	14.4 ± 3.8	45.2 ± 4.1	0.0001
Insulin OST 60 min (μIU/mL)	22.9 ± 4.0	100.3 ± 4.3	<0.0001
Body weight (kg)	547.1 ± 19.3	547.0 ± 21.3	1.0
Body condition score^a^	5.7 ± 0.2	7.8 ± 0.2	<0.0001
Age (years)*	14.0 ± 1.5	14.9 ± 2.0	0.7

ID, insulin dysregulated horses; OST, oral sugar test. Data are presented as least square means ± standard error of the mean. n = 8 non-ID and n = 7 ID horses. ^a^Body condition score was determined according to Henneke et al. (11). ^*^Age was not known for all horses due to limited history on horse donations (n = 7 known for non-ID, n = 4 known for ID group). P < 0.05 indicates differences between ID and control group.

Horses were housed in individual stalls (3.5 × 3.5 m) overnight and turned out in pairs into outdoor dry lot paddocks for ~6 h/day. They had access to water, salt and mineral blocks in the outdoor paddocks, but no feed was provided during turnout. Timothy hay was offered in hay nets in the individual stalls at 1.25% of BW (as fed) per day. Based on the estimated digestible energy (DE) consumed from this amount of hay, each horse then received a variable amount of a custom-made concentrate mixture so that the total diet provided 120% of the daily DE requirements for average maintenance (12). The concentrate consisted of 11% soybean oil, 44% whole oats and 45% beet pulp shreds (Table 2). Additionally, 100 g/day of a commercially available vitamin-mineral pellet (Cavalor Support, Cavalor Feeds and Supplements, Drongen, Belgium) was mixed into the daily meals. This dietary regimen was designed to meet or exceed all nutrient needs for mature, idle horses with “average” maintenance requirements (12). Concentrate meals were provided twice a day at 08:00 and 16:00 and the daily allotment of hay was given in the afternoon. Leftover feed (weighed back after morning and evening meals) and hay (weighed back every morning) was recorded for feed intake quantification. Feed intake was averaged over a 7-day period and comparisons were made between “pre bromo” (average feed intake for 7 days prior to start of bromocriptine treatment), “week 1” (average feed intake on days 1-7 of bromocriptine treatment) and “week 2” (average feed intake on days 7–14 of bromocriptine treatment). All horses were adapted to housing and diets for at least 6 weeks prior to any measurements.

**Table 2:** Nutrient composition of the daily grain mixture and timothy hay on dry matter basis.

**Nutrient**	**Grain mixture**	**Timothy hay**
	**% of DM**	
DE (Mcal/kg)^*^	3.1	2.14
Crude protein	8.9	7.75
Acid detergent fiber	18.4	38.2
Neutral detergent fiber	32.4	61.7
Water-soluble carbohydrates	6.5	9.9
Ether-soluble carbohydrates	6.0	8.4
Starch	20.6	1.7
Non-fiber Carbohydrates	49	21.2
Calcium	0.55	0.3
Phosphorus	0.25	0.3
Magnesium	0.19	0.14
Potassium	0.34	2.46
Sodium	0.011	0.006
	**PPM**	
Iron	350	151.5
Zinc	22	32.5
Copper	5	6.5
Manganese	54	32.5
Molybdenum	0.4	1.15

* *DE calculated value (13)*.

Body condition score (BCS) and BW were assessed at the beginning and end of the study. BCS was determined on a 1–9 scale (11) by two independent scorers, blinded to the experimental design.

### Experimental Procedures

A 14-day repeated measures design was used to determine the effects of bromocriptine administration on insulin sensitivity and circulating concentrations of different blood metabolites. Due to barn space restriction, 8 (*n* = 4 ID and *n* = 4 control) and 7 (*n* = 3 ID and *n* = 4 control) horses were studied in the first and second experimental blocks, respectively, with blocks separated by 2 months (September and December). After an initial 2-day sample collection period (days −1 and 0; hereafter defined as “Pre Bromo”), all horses received the bromocriptine treatment on days 1, 4, 7, 10, and 13 followed by a second sample collection period (days 14 and 15; hereafter defined as “Post bromo”). On the morning of Day 7, an additional blood sample was collected *via* jugular venipuncture before horses received their morning meal.

The day before each sample period, all horses were weighed and an area over the jugular vein was clipped. For all testing procedures, concentrate meals were withheld from 19:00 the night before each sample day but horses had access to hay until 2 h prior to the start of each procedure. Throughout all testing procedures horses had access to water. Immediately following completion of each procedure, all horses were allowed to eat their morning concentrate meals and a portion of their hay before being turned out for the remainder of the day.

Blood samples were collected at predetermined times for each procedure (see below) and collected into heparinized vacutainer tubes. One additional blood sample was collected in to an EDTA-coated tube prior to initiation of each procedure for analysis of fat metabolites. All blood samples were stored on ice and plasma harvested after centrifugation (1,500 × g for 10 min) when testing procedures were completed. Plasma samples were stored at −20°C until further analysis.

#### CGIT Procedures

On days −1 and 14, insulin sensitivity was assessed from a combined glucose insulin tolerance test (CGIT) administered as previously described (14). Briefly, intravenous catheters were aseptically placed in the jugular vein after which horses were allowed to recover for ~30 min before starting the procedures. Two baseline blood samples (10 mL) were collected at −15 and 0 min and blood glucose levels measured immediately using a handheld glucometer, after which horses were rapidly (<1 min) infused intravenously with a bolus of sterile 50% dextrose solution that provided 150 mg/kg BW. Immediately following, a sterile intravenous insulin injection was given at a rate of 0.1 U/kg BW (diluted in ~3 mL of isotonic saline solution). Additional blood samples (10 mL) were collected 1, 5, 15, 35, 45, 60, 75, 90, 105, 120, 135, and 150 min after the insulin injection. To monitor glycemia, blood glucose concentrations were determined from each collected sample throughout the procedure using a handheld glucometer. Besides occasional light sweating, no horses showed concerning signs of hypoglycemia and no emergency dextrose administration was needed during any of the procedures.

#### Feed Challenge Procedures

In further assessment of changes in insulin and glucose dynamics following bromocriptine treatment, each horse was subjected to a feeding challenge on days 0 and 15. On the morning of each test, two baseline blood samples were collected by jugular venipuncture at −30 and 0 min after which each horse received an amount of whole oats (52.8% starch on DM basis) that provided 1 g starch/ kg BW. This amount of cereal grain starch has previously shown to elicit a glycemic and insulinemic response in horses (15). Additional blood samples were collected by jugular venipuncture 60, 120, 180, 240, and 300 min after the meal was administered. Time to completion of the meal and any leftover grain was recorded.

#### Bromocriptine Preparation and Administration Procedures

Bromocriptine mesylate (Santa Cruz Biotechnology, Dallas, TX) was prepared and administered as previously described (16) and was reconstituted using 90% ethanol to achieve a final concentration of 13.6 mg/mL. The bromocriptine treatments were given as intramuscular injections on alternate sides the neck every 3 days for 14 days (five injections total) at a rate of 0.1 mg/kg BW. This dosing regimen has been shown to maintain sustained reduction in prolactin in cattle (16–18). Similar doses have also been used in horses to study the effects of bromocriptine on plasma prolactin and progesterone (19) and thyroid hormone concentrations (20). Each injection was given in the morning prior to turn out. Expecting mild discomfort from the injections, horses were lightly sedated with ~2–3 mL of i.v. xylazine hydrochloride (Anased, Llyod Inc., Shenandoah, IA) and placed into holding stocks during the first two injections. When no adverse behavioral reactions were noted for any horse, the remainder of injections were given in the stall without sedation without any issue. Occasional mild swelling on the neck was observed but subsided within a few days and caused no significant discomfort to any of the horses.

All procedures were approved by the Institutional Animal Care and Use Committee at the University of Kentucky.

### Sample Analyses

#### Plasma Glucose and Insulin Analyses

Whole blood glucose concentrations during the CGIT procedure were measured immediately after collection using a handheld glucometer (Accu-Chek Aviva, Roche Diagnostic, Indianapolis, IN). Plasma glucose concentrations from samples collected during the feed challenge test were determined with an enzymatic assay (Konelab i 20XTi, Thermo Electron Corp., Waltham, MA). Plasma insulin concentrations were determined with a commercially available radioimmunoassay kit (PI-12K, Millipore Sigma, Burlington, MA) with has previously been validated in horses (21). All plasma samples were run in duplicate within a single assay. Average intra-assay variation was 1.3 and 5.1% for glucose and insulin, respectively.

#### Plasma Lipid Analyses

Plasma triglycerides and cholesterol concentrations were measured with an enzymatic assay (Konelab i 20XTi, Thermo Electron Corp., Waltham, MA) using commercially available reagents [Infinity cholesterol (TR13421) and triglyceride (TR22421) reagents, Thermo Fisher, Waltham, MA] and standards [Stabio cholesterol standard (SB1012030), Thermo Fisher, Waltham, MA; triglyceride standard (T7531STD), Pointe Scientific, Canton, MI]. Plasma HMW adiponectin was measured using a commercially available kit (EZHWAM-65, Millipore Sigma, Burlington, MA) previously validated for use in lean and obese horses (22). Average intra-assay variation for plasma adiponectin was 3.14%.

#### Plasma Prolactin Analysis

Plasma prolactin concentrations were determined in duplicate using a radioimmunoassay previously validated for equine samples (23). Intra- and interassay variation and levels of detection were 7%, 12%, and 0.2 ng/mL, respectively.

#### Analysis of the CGIT Results

Analysis of the CGIT results was conducted as previously described (24). Briefly, “positive phase” is defined as the period of the CGIT procedure during which glucose concentrations are elevated above baseline (*t* = 0 min) concentrations, not including glucose recovery (i.e., period where glucose concentrations rise above baseline after reaching nadir concentrations). The positive phase duration (defined as “time in positive phase”) was calculated as the time (min.) between the start of the CGIT (*t* = 0 min) until the timepoint of lowest measured glucose concentration in the positive phase (defined as “lowest positive phase glucose concentrations”). Time until nadir was calculated as the time between the start of the CGIT (*t* = 0) until the time of lowest measured glucose concentration during the entire CGIT procedure. Positive phase glucose clearance rate was calculated by dividing the difference between peak and lowest positive phase glucose concentrations by the difference in time between positive phase duration and time to reach peak glucose concentrations. Delta *t* = 45 min was calculated as the difference between glucose concentrations at 45 min and baseline (*t* = 0) glucose concentrations. Horses were considered insulin dysregulated when the positive phase duration >45 min or if plasma insulin concentrations were >20 μIU/mL at 75 min (14). Area under the glucose response curve (AUC) was evaluated for the positive phase (i.e., area under the positive response curve until baseline glucose concentrations were reached). The negative phase (i.e., glucose levels below baseline) or recovery phase (i.e., glucose concentrations above baseline near the end of the CGIT) were not considered in the final analysis as it would be impossible to distinguish between glucose clearance and hepatic glucose production at this point.

### Statistical Analyses

Area under the glucose response curve for the CGIT were calculated using the trapezoidal method with commercially available software (GraphPad Prism). All data were analyzed using mixed procedures in SAS 9.4 statistical software (SAS Institute, Cary, NC) with significance considered at *P* ≤ 0.05 and trends considered when 0.05 < *P* ≤ 0.10. Screening data (Table 1) were analyzed using a one-way ANOVA to assess differences between groups (i.e., ID vs. non-ID) at the start of the study. Variables measured over time were analyzed using repeated measures two-way ANOVA with appropriate variance-covariance structures chosen for each variable based on lowest AICC fit statistics, and the kr2 adjustment for degrees of freedom. Insulin status (“INS”, i.e., ID vs. non-ID horses), sample period (“Bromo”, i.e., pre bromo vs. post bromo; pre bromo vs. week 1 vs. week 2; or Pre bromo vs. Day 7 vs. Post bromo measures), and their interactions were considered fixed effects, with sample period as the repeated measure and horse as the random subject. Block was considered a random effect in the model. The repeated measures model used for the feed challenge data included insulin status, sample period (i.e. pre vs. post bromo), time point (before and 60, 120, 180, 240, and 300 min post feeding) and their interactions as fixed effects, with time as the repeated measure and horse nested within sample period as the random subject. Block was considered a random effect in the model. Data for horses that did not consume their test meal within 60 min were removed from the final data set. This resulted in *n* = 7 ID and *n* = 6 non-ID for pre bromocriptine measures, and *n* = 4 ID and *n* = 6 non-ID comparisons for post treatment measures. However, as there was no significant sample period by insulin status interaction, data were combined to look at main effects of bromocriptine (i.e., *n* = 13 pre vs. *n* = 10 post) and insulin status (i.e., *n* = 12 non-ID vs. *n* = 11 ID). For all data, when fixed effects were significant, least square means were compared using the SAS pdiff test.

Heterogeneity of variance was assessed for each response variable by evaluating the AICC criterion that resulted from alternately setting the “group” option within the repeated statement equal to each of the main effects and their interaction and comparing that to the homogenous model without a “group” specification. In each case, the model with the smallest AICC value was chosen as the best-fit model, resulting in defining either “insulin status” or “sample period” as the source of heterogeneity in the covariance structure. Studentized residuals for all response variables were evaluated graphically (using Q/Q plots) and with the SAS univariate procedures to ensure adherence to normality assumptions. Due to non-normal distribution, prolactin data were transformed to their square root and insulin (CGIT and feed challenge data) and triglyceride data to their natural logarithmic values before analysis. Transformed data are presented as backtransformed data ± the 95% confidence intervals where appropriate.

All other data are presented as least square means and standard error of the means unless otherwise indicated. As the statistical significance of treatment comparisons are based on the standard error of the difference (SED), these values are also reported in the tables for reference.

## Results

### Body Weight and BCS

ID horses had a higher BCS compared to non-ID horses (*P* < 0.0001), but BW was not different between groups (*P* = 0.9, Table 3). Bromocriptine treatment caused a small decrease in BW in both groups (*P* = 0.03) and slightly increased the BCS of non-ID horses (*P* = 0.04) but not that of the ID horses (*P* = 0.4).

**Table 3:** Changes in body weight and body condition score following bromocriptine treatment in ID and non-ID horses.

	**Non-ID**			**ID**			**SED bromo**	***P*** **-values**		
	**Pre bromo**	**Post bromo**	SED INS*Bromo	**Pre bromo**	**Post bromo**	SED INS*Bromo		**Bromo**	**INS**	INS*Bromo
BW (kg)	547.7± 18.6^a^	546.2 ± 18.6^b^	NA	547.0 ± 19.9^a^	541.4 ± 19.9^b^	NA	1.5	0.03	0.9	0.2
BCS*	5.7 ± 0.55^a^	6.0 ± 0.55^b^	0.12	7.8 ± 0.56^c^	7.7 ± 0.56^c^	0.12	NA	0.3	<0.0001	0.04

BW, body weight; ID, insulin dysregulated horses; INS, effect of insulin status; Bromo, effect of bromocriptine treatment; Data are presented as least square means ± standard error of the mean. Standard error of the differences (SED) is also provided for reference. SED INS^*^Bromo is the SED for the pre vs. post bromo comparison within each insulin status group. n = 8 non-ID and n = 7 ID horses.

^*^Body condition score was determined on a 1–9 scale by two independent scorers blinded to the experimental design (11).

^a,b,c^*Indicates significant differences between and within group (P ≤ 0.05)*.

### Daily Feed Intake

There was no significant (*P* ≥ 0.14) interaction between bromocriptine and insulin status for any of the measurements.

#### Effect of Insulin Status

Daily hay intake was higher (*P* = 0.02) in the ID horses compared to non-ID horses (10.8 vs. 12.4 g/kg BW for non-ID and ID, respectively) resulting in greater (*P* = 0.02) DE intake from hay (Table 4). There was no effect of insulin status or interactions with insulin status on grain intake, DE intake for grain or total DE intake (*P* ≥ 0.3).

**Table 4:** Effect of bromocriptine treatment and insulin status on daily feed and digestible energy intake (g/kg BW) on an as fed basis.

	**Effect of bromocriptine**				**Effect of insulin status**			***P*** **-values**	
	**Pre bromo**	**Week 1 bromo**	**Week 2 bromo**	**SED**	**Non-ID**	**ID**	**SED**	**Bromo**	**INS**
**Feed intake (g/kg BW)**									
Hay	11.32 ± 0.32^a^	11.51 ± 0.32^a^	11.99 ± 0.32^b^	0.21	10.8 ± 0.41	12.41 ± 0.43	0.60	0.03	0.02
Concentrate	4.73 ± 0.29^a^	2.75 ± 0.29^b^	2.45 ± 0.29^b^	0.32	3.56 ± 0.3	3.07 ± 0.32	0.44	<0.0001	0.3
**DE intake (kcal/kg BW)**									
Hay	22.52 ± 0.65^a^	22.89 ± 0.65^a^	23.85 ± 0.65^b^	0.43	21.53 ± 0.82	24.65 ± 0.87	1.20	0.04	0.02
Concentrate	13.30 ± 0.80^a^	7.73 ± 0.80^b^	6.84 ± 0.80^b^	0.89	9.98 ± 0.84	8.60 ± 0.90	1.24	<0.0001	0.3
Total	35.86 ± 0.97^a^	30.66 ± 1.51^b^	30.73 ± 1.48^b^	0.98	31.51 ± 1.52	33.32 ± 1.60	1.95	0.0002	0.4

*ID, insulin dysregulated horses; INS, effect of insulin status; Bromo, effect of bromocriptine treatment; Data are presented as least square means ± standard error of the mean. Standard error of the differences (SED) is also provided for reference. SED for effects of bromocriptine is the average SED for each time point comparison. n = 8 non-ID and n = 7 ID horses*.

^a,b^*Illustrates significant differences between specific sample periods (P ≤ 0.05)*.

#### Effect of Bromocriptine Treatment

Daily hay consumption and DE intake from hay in the first week of bromocriptine treatment was not different (*P* > 0.3) from pre-treatment measurements but slightly increased from Week 1 to Week 2 (*P* = 0.02; Table 4). Daily grain consumption and DE intake from grain decreased during the first week of bromocriptine treatment (*P* < 0.0001) and remained low in Week 2 (*P* < 0.0001). Consequently, total daily DE intake decreased in Week 1 (*P* < 0.0001) and remained lower (*P* = 0.0002) than pre-treatment values in Week 2.

### CGIT

There was no significant (*P* ≥ 0.2) interaction between bromocriptine and insulin status for any of the measurements.

#### Effect of Insulin Status

Peak glucose concentration following infusion tended (*P* = 0.10) to be higher in ID horses compared to non-ID horses (222.2 vs. 206.6 mg/dL, respectively). Glucose concentration at *t* = 45 min was higher (*P* = 0.001) in the ID horses compared to non-ID horses and did not return to baseline at *t* = 45 min in the ID group, resulting in a positive delta (i.e., *t* = 45 minus *t* = 0 min glucose concentrations, *P* = 0.0004). ID horses had higher (*P* = 0.0002) insulin concentrations at *t* = 75 min compared to non-ID horses (37.05 vs. 8.78 μIU/mL, respectively) (*P* = 0.10; Table 5). ID horses had slower (*P* = 0.02) glucose clearance rates compared to non-ID horses resulting in a longer (*P* = 0.03) time in positive phase and larger (*P* = 0.02) AUC. Consequently, time to reach nadir glucose concentrations was longer (*P* = 0.02) in ID horses compared to non-ID horses. Lastly, nadir glucose concentrations were higher (*P* = 0.02) in ID horses compared to non-ID horses. There was no effect (*P* ≥ 0.6) of insulin status on baseline glucose concentrations or lowest positive phase glucose concentrations.

**Table 5:** Effect of bromocriptine and insulin status on CGIT measurements.

	**Effect of bromocriptine**			**Effect of insulin status**			***P*** **-values**	
	**Pre bromo**	**Post bromo**	**SED**	**Non-ID**	**ID**	**SED**	**Bromo**	**INS**
Basal glucose conc. (mg/dL)	96.65 ± 1.63	97.45 ± 1.63	1.94	97.75 ± 1.797	96.36 ± 1.92	2.63	0.7	0.6
Peak glucose conc. (mg/dL)	210.63 ± 5.98	217.15 ± 5.98	5.45	206.56 ± 6.57	221.22 ± 7.0	8.41	0.2	0.1
Glucose 45 min conc. (mg/dL)	81.69 ± 7.53	92.823 ± 7.53	5.04	70.87 ± 7.93	103.65 ± 8.21	7.68	0.05	0.001
Delta 45 glucose conc. (mg/dL)*	−14.96 ± 7.22	−4.63 ± 7.22	4.63	−26.87 ± 7.59	7.28 ± 7.84	7.15	0.04	0.0004
Insulin 75 min conc. (μIU/mL)	14.59 (8.49–25.07)	20.27 (12.72–32.28)	0.08	8.78 (7.0–11.03)	37.05 (25.11–54.65)	0.22	0.001	0.0002
Glucose clearance rates in positive phase (mg/dL/min)	4.60 ± 0.65	3.48 ± 0.65	0.42	5.33 ± 0.77	2.74 ± 0.82	1.0	0.02	0.02
Positive phase glucose AUC (mg/dL/min)	1,588.10 ± 339.79	1,789.34 ± 280.56	171.93	1,066.40 ± 256.34	2,311.04 ± 438.35	396.17	0.2	0.02
Time in positive phase (min)	39.51 ± 8.90	50.67 ± 8.03	4.69	24.37 ± 4.03	65.81 ± 14.96	14.66	0.04	0.03
Lowest positive phase glucose conc. (mg/dL)	102.39 ± 1.611	99.93 ± 1.61	1.86	101.75 ± 1.611	100.57 ± 1.92	2.63	0.2	0.7
Nadir glucose conc. (mg/dL)	69.92 ± 4.44	77.44 ± 4.44	4.06	64.25 ± 4.93	83.11 ± 5.27	6.47	0.09	0.01
Time until nadir (min)	78.28 ± 9.18	94.75 ± 9.45	5.74	70.31 ± 7.71	102.72 ± 12.56	10.98	0.01	0.02

ID, insulin dysregulated horses; INS, effect of insulin status; Bromo, effect of bromocriptine treatment; Data are presented as least square means ± standard error of the mean, with exception of insulin concentrations which are presented as backtransformed means ± 95% confidence interval. n = 8 non-ID and n = 7 ID horses. Standard error of the differences (SED) is also provided for reference. *Delta = t = 45 minus t = 0 min glucose concentrations.

#### Effect of Bromocriptine Treatment

Glucose clearance rates were decreased in all horses (*P* = 0.02) after 14 days of bromocriptine treatment resulting in a longer (*P* = 0.04) time in positive phase (Table 5). Consequently, *t* = 45 min glucose concentrations were higher (*P* = 0.05) post bromocriptine treatment, resulting in a smaller delta (*P* = 0.04). Additionally, time to reach nadir took longer (*P* = 0.01) and nadir glucose concentrations tended to be higher (*P* = 0.09) after bromocriptine treatment. Bromocriptine administration for 14 days also increased (*P* = 0.001) insulin concentrations at *t* = 75 min. A positive phase time >45 min and insulin concentrations > 20 μIU/mL post treatment indicates that 14 days of bromocriptine treatment reduced insulin sensitivity. There was no effect (*P* ≥ 0.2) of bromocriptine treatment on basal, peak or lowest positive phase glucose concentrations or AUC.

### Feed Challenge

There was no effect of bromocriptine (*P* = 0.6) or insulin status (*P* = 0.8) on time to finish the oats meal. Average meal consumption time was 20 ± 3 min for all horses. There was no significant interaction between bromocriptine treatment and insulin status or 3-way interaction with time for postprandial plasma glucose or insulin responses (*P* ≥ 0.2).

#### Effect of Insulin Status

**Figure 1:**
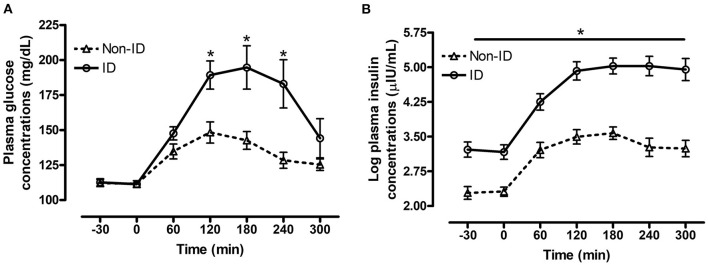
Effect of insulin status on postprandial glucose and insulin responses to feeding a meal of whole oats. Postprandial plasma glucose and log insulin concentrations following consumption of a meal of whole oats providing 1 g starch/kg BW in non-ID and ID horses. **(A)** Effect of insulin status (INS* time interaction *P* = 0.02) on glucose concentrations (mg/dL) at baseline −30, 0 min and 60, 120, 180, 240, and 300 min post feeding. **(B)** Effect of insulin status (INS* time interaction *P* = 0.04) on log insulin concentrations (μIU/mL) at baseline (−30, 0 min) and 60, 120, 180, 240, and 300 min post feeding. Dotted line with triangles: measurements in non-ID horses (*n* = 12); Solid line with circles: measurements in ID horses (*n* = 11). Data of horses that did not consume their meal within 60 min were removed from the final analysis. Data are presented as least square means ± standard error of the mean. *Indicates differences (*P* ≤ 0.05) between non-ID and ID horses for plasma metabolite concentrations at specific timepoints. ID, insulin dysregulated horses; INS, effect of insulin status.

There was a significant interaction between insulin status and time for both plasma glucose (*P* = 0.02, Figure 1A) and insulin (*P* = 0.04, Figure 1B) concentrations post consumption of the oats meal. ID horses had higher glucose concentrations at 60 (*P* = 0.09), 120 (*P* = 0.006), 180 (*P* = 0.009) and 240 min (*P* = 0.01) compared to non-ID horses. Additionally, postprandial insulin concentrations were higher (*P* ≤ 0.0004) in ID horses compared to non-ID horses at all timepoints.

#### Effect of Bromocriptine Treatment

**Figure 2:**
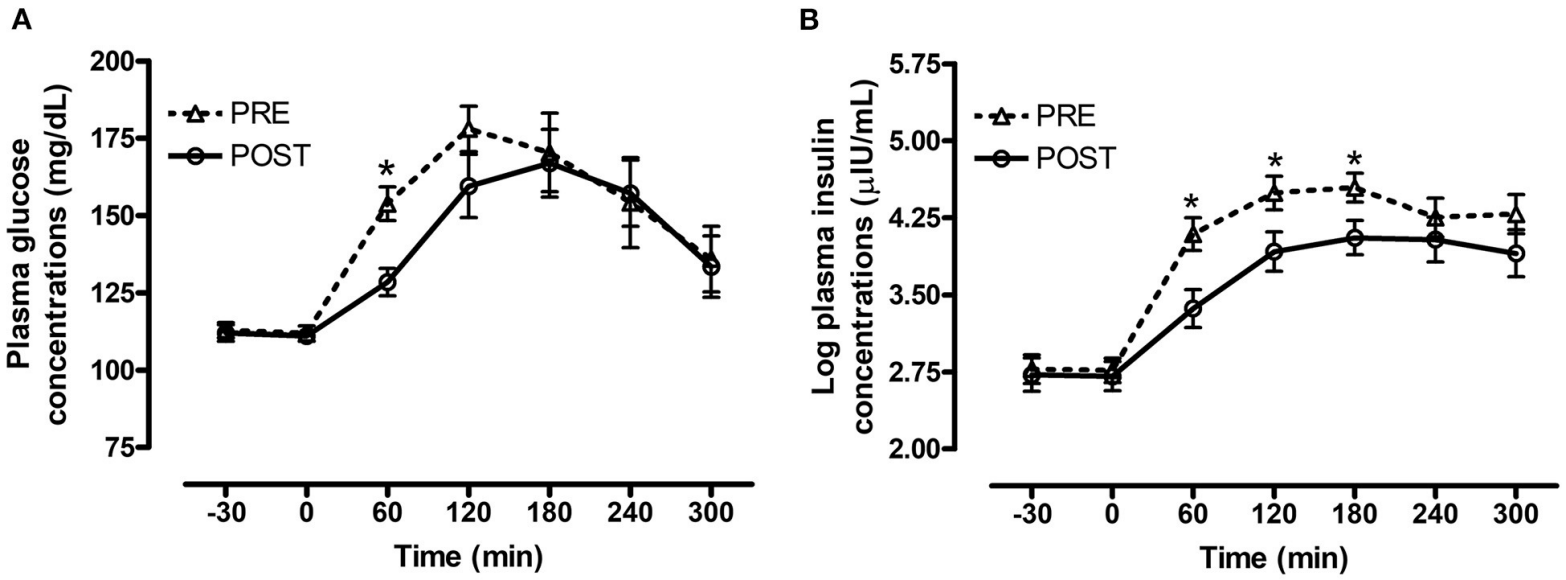
Effect of bromocriptine on postprandial glucose and insulin responses to feeding a meal of whole oats. Postprandial plasma glucose and log insulin concentrations following consumption of a meal of whole oats providing 1 g starch/kg BW before and after 14 days of bromocriptine treatment. **(A)** Effect of bromocriptine (bromo* time interaction *P* = 0.008) on glucose concentrations (mg/dL) at baseline (-30, 0 min) and 60, 120, 180, 240, and 300 min post feeding. **(B)** Effect of bromocriptine (bromo* time interaction *P* = 0.001) on log insulin concentrations (μIU/mL) at baseline (−30, 0 min) and 60, 120, 180, 240, and 300 min post feeding. Dotted line with triangles: measurements before start of bromocriptine treatment (*n* = 13); Solid line with circles: measurements after 14 days of bromocriptine treatment (*n* = 10). Data of horses that did not consume their meal within 60 min were removed from the final analysis. Data are presented as least square means ± standard error of the mean. *Indicates differences (*P* ≤ 0.05) between pre and post bromo plasma metabolite concentrations at specific timepoints. ID, insulin dysregulated horses; INS, effect of insulin status.

There was a significant interaction between bromocriptine and time for plasma glucose (*P* = 0.008, Figure 2A) and insulin (*P* = 0.001, Figure 2B) concentrations post consumption of the oats meal. Bromocriptine treatment decreased postprandial glucose concentrations at 60 min (*P* = 0.003) and decreased insulin concentrations at 60 (*P* = 0.007), 120 (*P* = 0.04) and 180 min (*P* = 0.04) post feeding.

### Plasma Metabolites

There was no significant (*P* ≥ 0.6) interaction between bromocriptine and insulin status for any of the measurements.

#### Effect of Insulin Status

ID horses had higher basal plasma insulin (*P* = 0.01) and triglyceride (*P* = 0.02) concentrations, but lower adiponectin (*P* = 0.05) concentrations compared to non-ID horses (Table 6). There was no effect of insulin status on cholesterol or prolactin concentrations (*P* ≥ 0.6).

**Table 6:** Effect of bromocriptine and insulin status on plasma metabolites.

	**Effect of bromocriptine**				**Effect of insulin status**			***P*** **-values**	
	**Pre bromo**	**Day 7 bromo**	**Post bromo**	**SED**	**Non-ID**	**ID**	**SED**	**Bromo**	**INS**
Prolactin conc. (ng/mL)	1.74 (0.9–2.9)^a^	0.29 (0.09–0.6)^b^	0.20 (0.1–0.4)^b^	0.16	0.64 (0.3–1.2)	0.56 (0.2–1.0)	0.13	0.0002	0.6
Cholesterol conc. (mmol/L)	1.82 ± 0.3^a^	1.57 ± 0.3^b^	1.44 ± 0.3^b^	0.15	1.64 ± 0.3	1.58 ± 0.3	0.23	0.1	0.8
Adiponectin conc. (μg/mL)	24.33 ± 3.4^a^	NA	26.88 ± 3.4^b^	0.99	31.55 ± 4.2	19.66 ± 4.5	5.46	0.02	0.05
Basal insulin conc. (μIU/mL)	20.50 ± 3.1	21.40 ± 3.1	19.97 ± 3.1	1.70	10.17 ± 1.1	31.08 ± 5.8	5.92	0.7	0.01
Triglycerides conc. (mmol/L)	19.16 (14.0–26.2)	19.17 (14.0–26.3)	21.86 (16.0–29.9)	1.16	15.37 (12.9–18.3)	27.09 (21.0–34.9)	0.20	0.6	0.02

*ID, insulin dysregulated horses; INS, effect of insulin status; Bromo, effect of bromocriptine treatment; NA, Not applicable, data not collected at this timepoint. Data are presented as least square means ± standard error of the mean, with exception of prolactin and triglyceride concentrations which are presented as backtransformed means ± 95% confidence interval. Standard error of the differences (SED) is also provided for reference. SED for effects of bromocriptine is the average SED for each time point comparison. n = 8 non-ID and n = 7 ID horses*.

^a,b^*Illustrates significant differences between specific sample periods (P ≤ 0.1)*.

#### Effect of Bromocriptine Treatment

As expected, bromocriptine dramatically (i.e., 6-fold) decreased circulating plasma prolactin (*P* < 0.0002) concentrations in all horses and tended to decrease cholesterol (*P* = 0.10) levels, both of which reached nadir after 7 days of treatment (Table 6). Prolactin and cholesterol concentrations remained lower than pretreatment values for the remainder of the study. Additionally, 14 days of bromocriptine treatment increased (*P* = 0.02) adiponectin concentrations. There was no effect (*P* ≥ 0.6) of bromocriptine on basal insulin or triglyceride concentrations.

## Discussion

Ergot alkaloids (EA) are known to affect metabolic pathways in human and animal species (25). While consumption of EA is most often associated with a negative impact on animal health (e.g., reduced growth in cattle, negative impact on hemodynamics, fetal loss in horses, etc.), certain synthetic EA have also been shown to have beneficial physiological and potential therapeutic effects in humans and horses (26–28). Bromocriptine, a well-characterized synthetic EA and D2-receptor agonist, has shown to improve glucose homeostasis leading to its use as a pharmaceutical agent to improve insulin sensitivity in humans (29, 30). Considering insulin dysregulation (ID) remains a prevalent clinical condition in horses, it was of interest to evaluate the effects of bromocriptine on insulin dynamics. In contrast to our hypothesis and data in humans and rodents, 14 days of intramuscular bromocriptine administration reduced insulin sensitivity in all horses, regardless of their insulin status. This suggests that the regulation of glucose and insulin dynamics might be different in horses compared to humans. Additionally, the structural and functional similarities between bromocriptine and ergovaline (29, 31, 32), the predominant EA found in endophyte-infected fescue, now raises the question of potential implications on insulin metabolism for horses grazing ergot alkaloids (EA).

### Effect of Bromocriptine

Bromocriptine administration reduced circulating prolactin concentrations in all horses, indicative that our treatment protocol was successful. Sharing a high degree of structural homology with dopamine, the inhibitory effect of bromocriptine on pituitary prolactin secretion has been documented extensively in many species, including horses (18, 19, 33, 34). In accordance with recent data in cattle (17), prolactin levels in all horses sharply declined during the first week of bromocriptine treatment and remained at nadir throughout the entire study period. Bromocriptine also reduced plasma cholesterol concentrations and increased plasma adiponectin concentrations in all horses. A decrease in blood lipid concentrations is a common finding with bromocriptine treatment and is often accompanied by reduction in body fat stores and generally improved metabolic profile (30, 35, 36). Although no quantitative measures of body fat stores were made, body condition scores (i.e., a visual scoring system of general adiposity) of the non-ID horses were slightly decreased after 14 days of bromocriptine treatment. As dopamine receptor stimulation initiates a multitude of neuronal, hormonal and cellular signaling events in various tissues, the exact underlying mechanisms for bromocriptine-mediated effect on lipid metabolism cannot be delineated in the current study. Some of its effects could be mediated through inhibition of prolactin secretion, a hormone well-known to play a role in lipid metabolism (35, 37). Alternatively, recent reports illustrate bromocriptine directly modulates lipid-related signaling pathways through dopamine receptors in the liver and adipose tissues (38–40).

Improvements in insulin sensitivity with bromocriptine treatment in ID humans and rodents, is attributed in part to the typical increase in adiponectin concentrations following bromocriptine treatment (41). Adiponectin plays an essential role in the regulation of energy balance and insulin action and circulating levels are typically reduced in obese and insulin resistant individuals, including horses (42). Despite an increase in plasma adiponectin concentrations, insulin sensitivity was decreased in both ID and non-ID horses after 14 days of bromocriptine treatment. Previous work in both non-ID and ID horses reported no difference in insulin sensitivity after treatment with other dopamine agonists, including pergolide mesylate and cabergoline (6, 7). This suggest that the physiological effects of synthetic EA are not only species-specific, but might also depend on their affinity and action on particular dopamine receptors. While a decrease in insulin sensitivity was unexpected, it is in accordance with recent reports using bromocriptine (17) and fescue-derived EA (43) in growing cattle. Moreover, some studies in humans report a differential effect of bromocriptine between lean and obese individuals, and similarly found a reduction in insulin sensitivity in lean individuals in contrast to an improvement in the obese cohort (3, 44). Interestingly, these authors report that timing of bromocriptine administration relative to the natural circadian neuroendocrine rhythms might play a pivotal role on its metabolic effects, in particular in healthy individuals with normal dopamine activity. One study reported that bromocriptine-mediated improvement in insulin sensitivity was only measurable in lean individuals when treatment was administered in the evening, when dopamine levels naturally decline (3). Conversely, an obesity-related hypo-dopaminergic state, allowed for measurable improvements in insulin sensitivity regardless of timing of bromocriptine administration (3). Bromocriptine in the current study was administered in the morning, yet insulin sensitivity in the current study was decreased in both non-ID and the obese, ID horses. There is limited data on dopamine circadian rhythms in horses, however, one study reported peak blood dopamine concentrations in the evening, rather than in the morning in old horses (>21 years of age) (45). Although the horses in the current study were younger, it would seem bromocriptine administration occurred at the time of lowest dopamine levels. Alternatively, a study in over 1,000 non-ID individuals suggested that interactions between prolactin and insulin might shift with age, with a positive relationship seen in elderly humans and a negative relationship reported in younger participants (46). Circulating prolactin concentrations are higher in older horses compared to younger horses (47, 48), while insulin sensitivity typically seems to decrease with age in horses (49). It could therefore be speculated that low prolactin concentrations might in fact be associated with lower insulin sensitivity in horses. Taken together these data indicate that dopaminergic regulation of insulin sensitivity differs in horses compared to humans and rodents, warranting additional species-specific investigation, in particular with regards to horses grazing on endophyte-infected grass species.

Despite a decrease in peripheral insulin sensitivity, the insulin response following consumption of the high starch (oats) meal was lowered by bromocriptine in all horses. This was unexpected, as reduced insulin sensitivity is typically associated with increased pancreatic insulin secretion in response to an oral glycemic challenge. The discrepancy between these two measures of insulin metabolism is unclear. It could be speculated that bromocriptine might influence glucose absorption and metabolism. Studies in humans report similar findings, where peripheral insulin sensitivity was either decreased or unchanged, but the glycemic response to an oral glucose load was lowered with bromocriptine treatment (3, 30). Although exact mechanisms are not fully elucidated, it is suggested that bromocriptine specifically improves post-prandial glucose disposal by inhibiting hepatic glucose production, enhancing splanchnic glucose uptake enhancing glucose-mediated glucose uptake (30). Alternatively, bromocriptine is known to decrease gastric emptying and intestinal transit (50, 51). Given the observed time course response as well as decreased tissue insulin sensitivity, the most likely explanation is that postprandial plasma glucose appearance and time to reach peak concentrations were delayed due to a decrease in gastric emptying rather than an increase in glucose clearance. While the adverse effects on insulin sensitivity warrant further investigation, these data indicate that bromocriptine improved the glucose and insulin response to an oral glycemic challenge in both obese ID and lean non-ID horses.

Total daily DE intake decreased with bromocriptine treatment, which was mostly attributed to a decrease in concentrate consumption. To account for potential waste and refusals, the total daily ration provided 120% of energy required for maintenance. Consequently, despite decreased DE intake due to concentrate refusals, the total DE intake was still sufficient to meet maintenance energy requirements. Moreover, a decrease in energy intake would typically be associated with improvement of insulin sensitivity rather than a decline. Therefore, the observed metabolic and hormonal changes were likely related to a direct effect of bromocriptine and not attributed to decreased energy consumption. The anorexic effect of dopamine agonists, including bromocriptine and fescue-derived EA, is well-known in several animal species and was therefore not unexpected (52–55). The dopamine agonist, pergolide, which is commonly used in the treatment of equine pituitary pars intermedia dysfunction, has been shown to decrease appetite in horses (56). However, in contrast to previous reports where animals were only presented with a single food source, it became obvious in the current study that bromocriptine treatment seemed to alter feed preference rather than total feed intake. Grain intake decreased abruptly with the start of bromocriptine administration and remained low for the entire study. Hay intake, on the other hand, increased. Anecdotally, this did not seem to be caused by a decreased appetite, as horses continued to express behavior of excitement in anticipation of receiving their morning and evening meals. However, they refused or were reluctant to eat once presented with the concentrate. In accordance, an elegant study in rats showed that the dopamine D2 receptor agonist, quinpirole, specifically decreased the preference for highly palatable, energy-dense food constituents (57). It seems plausible that bromocriptine, being a D2 receptor agonist, caused a similar response leading to aversion for the more palatable, calorically dense concentrate but not hay. Further investigation of dopaminergic control of feed preferences in horses and how this might be impacted by dopamine agonists, including fescue-derived ergot EA, is warranted.

### Effect of Insulin Status

As expected, ID horses had a greater insulin response to consumption of a high starch (oats) meal and 2-fold slower glucose clearance rates during the CGIT compared to their non-ID counterparts. Additionally, ID horses had higher basal plasma insulin concentrations, higher average insulin concentrations at time = 75 min during CGIT (37 vs. 9 μIU/mL for non-ID horses), and a longer positive phase time (65 min compared to 24 min for non-ID horses). These data clearly confirm this cohort was hyperinsulinemic and insulin resistant. These data are in accordance with previous studies that have used the CGIT as a measure of peripheral insulin resistance (14). Plasma glucose concentrations following consumption of the high starch (oats) meal were higher in the ID horses at several timepoints compared to the non-ID horses, which is in accordance with previous work in horses (58). Considering starch intake was determined on a body weight basis and meal consumption time did not differ between groups, it is likely that postprandial plasma glucose clearance rates were slower in the ID horses due to the underlying insulin resistance. Alternatively, it has been suggested that glucose absorption might be enhanced in the ID horse. A previous study observed similar glucose responses between hyperinsulinemic (HI) and normoinsulinemic (NI) horses following an intravenous glucose challenge but a significantly greater glucose response in the HI horses compared to the NI horses after an oral glucose challenge (59). Moreover, human studies illustrate that glucose absorption is increased in obese people due to upregulation of the sodium-glucose co-transporter (51). Taken together, is plausible that enhanced glucose absorption contributed to the higher plasma glucose and concomitant insulin responses following the high starch (oats) meal in the ID horses.

Most ID horses in the current study were obese, as evident from an average BCS of 7.8. Elevated plasma triglycerides and lower adiponectin concentrations confirm abnormalities in the lipid profiles and insulin status in these horses. Having a typical inverse relationship with adiposity, hypoadiponectinaemia is common in obese and ID humans and horses (22, 42, 60). Because of its insulin-sensitizing actions (i.e., enhanced insulin signaling and fatty acid oxidation in insulin sensitive tissues), it is believed that lower circulating adiponectin levels are involved in the development of IR (61). Additionally, adiponectin is also known to decrease triglyceride concentrations, which explains why the observed hypoadiponectinaemia in the obese horses was associated with increased circulating triglyceride concentrations. Additionally, reduced insulin-mediated inhibition of hepatic triglyceride synthesis and release as well as changes in lipid and fatty acid transporters of insulin-sensitive tissues also may contribute to elevation of plasma triglyceride concentrations (62–64). Taken together, the ID horses were insulin resistant, hyperinsulinemic and had altered lipid profiles compared to non-ID horses. However, these metabolic aberrations did not affect the response to bromocriptine.

In conclusion, bromocriptine treatment for 14 days resulted in a decrease in insulin sensitivity in both non-ID and ID horses, which is in contrast with data in human and rodent models but similar to results in growing cattle. Conversely, bromocriptine treatment lowered the insulin response to an oral glycemic challenge in both ID and non-ID horses. Additionally, it was observed that bromocriptine might alter feed preferences in horses, away from calorie-dense feedstuffs. These results indicate that the physiological effects of ergot alkaloids might be different in horses compared to other species and warrants further species-specific investigation, in particular with regards to horses grazing endophyte-infected grasses.

## Data Availability Statement

The raw data supporting the conclusions of this article will be made available by the authors, without undue reservation.

## Ethics Statement

The animal study was reviewed and approved by University of Kentucky Institutional Animal Care and Use Committee.

## Author Contributions

KM and CL conception and design of research. CL, AB, EO, and KM performed experiment and lab analyses. CL, EV, and KM analyzed data and interpreted results of experiments. CL prepared figures and drafted manuscript. CL, KU, EV, and KM edited and revised manuscript. CL, KU, EV, AB, EO, JK, and KM approved final version of manuscript. All authors contributed to the article and approved the submitted version.

## Funding

The information reported in this paper is part of a project of the Kentucky Agricultural Experiment Station and is published with the approval of the Director. This project was supported by USDA-Agricultural Research Service National Program 101, Food Animal Production. Mention of trade name, proprietary product, or specified equipment does not constitute a guarantee or warranty by the University of Kentucky and does not imply approval to the exclusion of other products that may be available.

## Conflict of Interest

The authors declare that the research was conducted in the absence of any commercial or financial relationships that could be construed as a potential conflict of interest.

## Publisher's Note

All claims expressed in this article are solely those of the authors and do not necessarily represent those of their affiliated organizations, or those of the publisher, the editors and the reviewers. Any product that may be evaluated in this article, or claim that may be made by its manufacturer, is not guaranteed or endorsed by the publisher.

## Footnotes

^1^ https://sites.tufts.edu/equineendogroup/files/2020/09/200592_EMS_Recommendations_Bro-FINAL.pdf

^2^ https://sites.tufts.edu/equineendogroup/files/2021/12/2021-PPID-Recommendations-V11-wo-insert.pdf
